# Age, disability, and household composition of nurses and physicians who are not in the labor force

**DOI:** 10.1371/journal.pone.0247967

**Published:** 2021-02-26

**Authors:** Dan P. Ly

**Affiliations:** Division of General Internal Medicine and Health Services Research, David Geffen School of Medicine at UCLA, Los Angeles, California, United States of America; University of Washington, UNITED STATES

## Abstract

While several areas in the United States have asked nurses and physicians who are not in the labor force to return to help with the COVID-19 pandemic, little is known about the characteristics of these clinicians that may present barriers to returning. We studied age, disability, and household composition of clinicians not in the workforce using the American Community Survey from 2014 to 2018, a nationally-representative survey of US households administered by the US Census. Overall, we found that, for nurses and physicians not in the labor force, over three-quarters were 55 and over and about 15 percent had a disability. For female nurses and physicians not in the labor force, over half of those ages 20–54 had a child under 15 at home and over half of those ages 65+ had another adult 65 and over at home. These characteristics may present challenges and risks to returning.

## Introduction

Earlier on during the COVID-19 pandemic, several areas in the United States asked nurses and physicians who were not in the labor force to return [1]. As cases rose again, such requests were reconsidered [2]. However, national estimates of age, disability, and household composition of these clinicians not in the workforce are unknown, which may be important when considering clinical workforce capacity for recrudescences of the pandemic and for future pandemics. The presence of children may present challenges to returning due to closure of in-person schooling, being older and having other elderly adults at home may present risks to returning given the relationship between age and COVID-19-related morbidity and mortality [3], and those with a disability may be unable to return.

## Methods

We used data from the American Community Survey (ACS) 2014–2018 5-year file, which pools data to represent 5% of the population. The ACS is a nationally-representative, US Census Bureau-administered survey of US households. The survey had a response rate of 92% to 97% from 2014–2018 [4]. The survey is collected by Internet, mail, telephone, and personal-visit interviews.

Occupation was self-reported. Disability was self-reported and defined as having ambulatory difficulty (limited in basic physical activities such as walking), cognitive difficulty (trouble with processes such as remembering or making decisions), self-care difficulty, or independent living difficulty. We focused on nurses and physicians who reported not being in the labor force (not working or not seeking work). We excluded those younger than 20. We also excluded those who had a child in the past year as labor force participation status may reflect status immediately after childbirth. We estimated number of nurses and physicians not in the labor force. We then estimated, by profession and sex, the weighted number of clinicians in each age category (20–54; 55–64; 65+) with a disability, with child at home younger than 15, and with adult at home (not including themselves) 65 and older. We used ACS-provided replicate sampling weights and the complex survey modules in Stata (StataCorp), version 16.0, to account for the ACS’s complex survey design and to make nationally-representative estimates. The UCLA IRB determined that this was not human subjects research.

## Results

**Fig 1:**
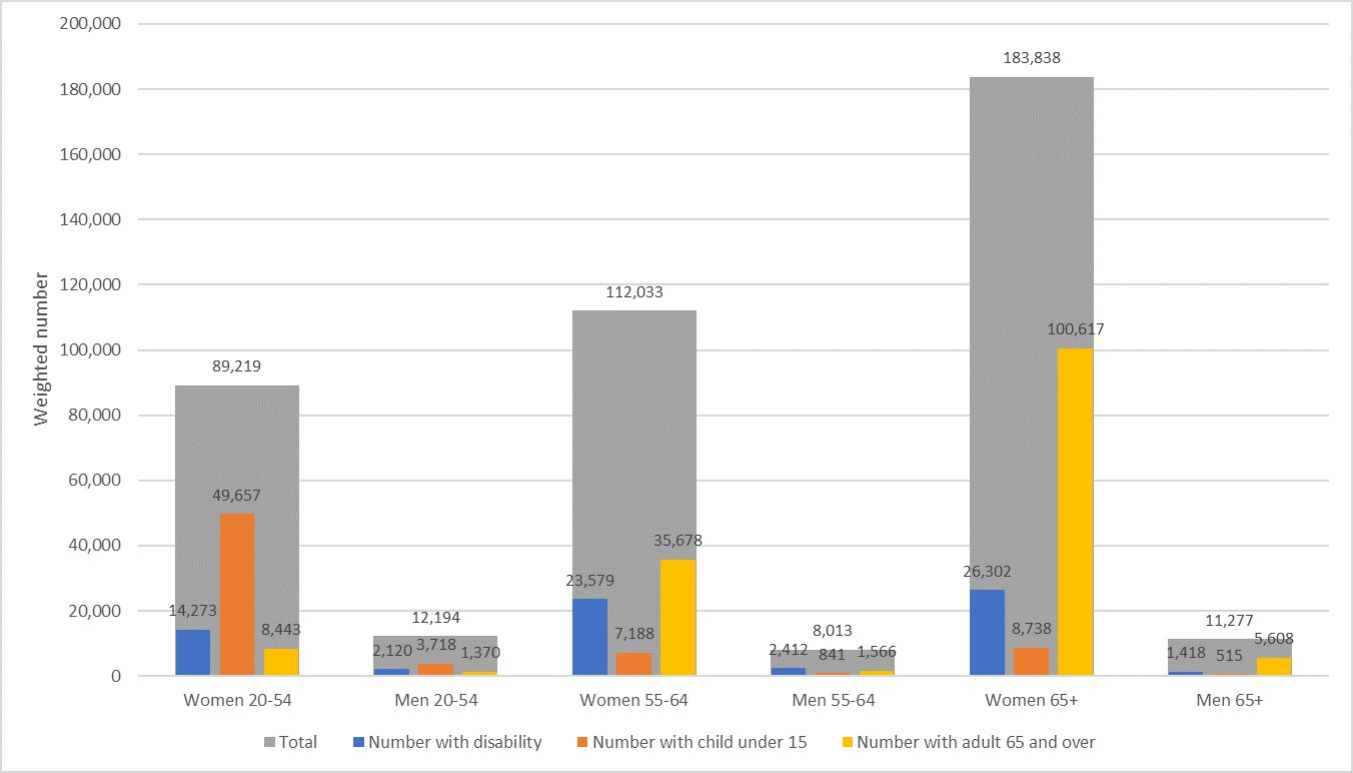
Weighted number of nurses not in labor force with a disability, with children under 15 in the household, and with other adults 65 and over in the household, 2014–2018. Note: Author’s calculation using American Community Survey (ACS) data from 2014–2018. The observation counts are weighted using ACS-provided replicate sampling weights to represent the US population. Occupation is self-reported. Disability is defined as having ambulatory difficulty (having a condition that substantially limits one or more basic physical activities, such as walking, climbing stairs, reaching, lifting, or carrying), cognitive difficulty (having a condition that leads to cognitive difficulties, such as learning, remembering, concentrating, or making decisions), self-care difficulty (having a condition that makes it difficult to take care of one’s own personal needs, such as bathing, dressing, or getting around inside the home), and independent living difficulty (having a condition that makes it difficult or impossible to perform basic activities outside the home alone).

**Fig 2:**
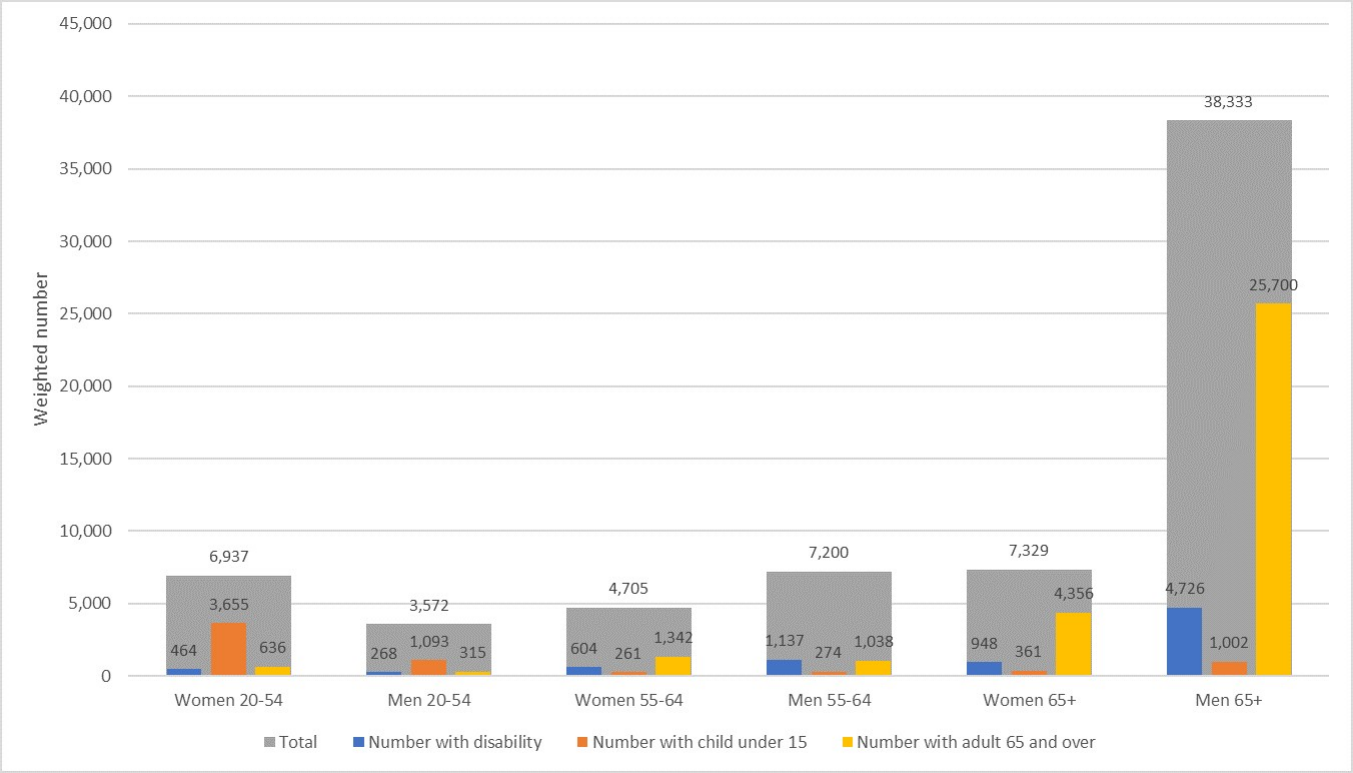
Weighted number of physicians not in labor force with a disability, with children under 15 in the household, and with other adults 65 and over in the household, 2014–2018. Note: Author’s calculation using American Community Survey (ACS) data from 2014–2018. The observation counts are weighted using ACS-provided replicate sampling weights to represent the US population. Occupation is self-reported. Disability is defined as having ambulatory difficulty (having a condition that substantially limits one or more basic physical activities, such as walking, climbing stairs, reaching, lifting, or carrying), cognitive difficulty (having a condition that leads to cognitive difficulties, such as learning, remembering, concentrating, or making decisions), self-care difficulty (having a condition that makes it difficult to take care of one’s own personal needs, such as bathing, dressing, or getting around inside the home), and independent living difficulty (having a condition that makes it difficult or impossible to perform basic activities outside the home alone).

Our sample included 189,521 nurses and 51,834 physicians; using ACS-provided sampling weights, this represents a total of 3,696,623 nurses and 981,248 physicians in the US. Approximately 11.3% (95% CI 11.1–11.4) of nurses and 6.9% (95% CI 6.7–7.2) of physicians were not in the labor force (Table 1), representing a total of 416,574 (95% CI 409,950–423,198) nurses and 68,076 (95% CI 65,404–70,748) physicians. Among those not in the labor force, approximately 76% of nurses and 85% of physicians were 55 and over. Among those not in the labor force 55 and over, approximately 17% of nurses and 13% of physicians had a disability. Among the 89,219 female nurses ages 20–54 not in the labor force, 55.7% (95% CI 53.7–57.6) had a child under 15 at home, while among the 195,115 nurses of both sexes ages 65+, 54.4% (95% CI 53.4–55.5) had another adult 65 and over at home (Fig 1). Among the 6,937 female physicians ages 20–54 not in the labor force, 52.7% (95% CI 45.8–59.6) had a child under 15 at home, while among the 45,662 physicians of both sexes ages 65+, 65.8% (95% CI 63.7–67.9) had another adult 65 and over at home (Fig 2).

**Table 1:** Sample characteristics of clinicians not in the labor force.

	Nurses	Physicians
	(n = 24,859)	(n = 4,093)
Women (%)	92.9 (92.5 to 93.2)	27.2 (25.8 to 28.6)
Men (%)	7.1 (6.8 to 7.5)	72.8 (71.4 to 74.2)
Mean Age	61.1 (61.0 to 61.3)	66.5 (66.1 to 66.9)
20–54 (%)	20.6 (20.1 to 21.1)	12.8 (11.8 to 13.8)
55–64 (%)	29.4 (28.8 to 30.0)	17.9 (16.7 to 19.1)
65+ (%)	50.0 (49.4 to 50.6)	69.3 (67.9 to 70.8)
With disability (%)	16.5 (16.1 to 17.0)	11.3 (10.3 to 12.2)
With child under 15 (%)	14.3 (13.9 to 14.8)	8.2 (7.4 to 9.1)
With adult 65 and over (%)	39.0 (38.4 to 39.6)	51.6 (50.0 to 53.1)

Note: Author’s calculation using American Community Survey (ACS) data from 2014–2018. Means and percentages are unweighted. Occupation is self-reported. Disability is defined as having ambulatory difficulty (having a condition that substantially limits one or more basic physical activities, such as walking, climbing stairs, reaching, lifting, or carrying), cognitive difficulty (having a condition that leads to cognitive difficulties, such as learning, remembering, concentrating, or making decisions), self-care difficulty (having a condition that makes it difficult to take care of one’s own personal needs, such as bathing, dressing, or getting around inside the home), and independent living difficulty (having a condition that makes it difficult or impossible to perform basic activities outside the home alone).

## Discussion

Using a nationally-representative sample, our findings suggest a majority of nurses and physicians in the US not in the workforce may be older, while those who are younger, particularly women, likely have children at home. If areas consider requesting volunteer nurses and physicians for recrudescences of the current pandemic or for future pandemics [2], known sex differences in division of household labor among clinicians may present challenges to younger clinicians returning [5], while a majority of clinicians available to return and their household members may be at higher risk of suffering poor outcomes from SARS-CoV-2 exposure or from exposure to pathogens with a similar age-related pattern of morbidity and mortality [3]. In addition, several disabilities, such as cognitive difficulties, may make returning to the workforce challenging for some clinicians, while certain disabilities, such as ambulatory difficulties, may not fully prevent other clinicians from returning. The barriers described here are not exhaustive and likely also apply to clinicians in the workforce. For example, many physicians in the workforce are also older and at higher risk of COVID-19-related morbidity and mortality if exposed [6]. Our results support continued efforts to provide childcare for health care workers, to provide workers with personal protective equipment, and to shift older clinicians to roles with less risk of SARS-CoV-2 exposure. Limitations of this study include reliance on self-reported occupation and lack of stated reason for not being in the labor force. Another limitation is the lack of specialty for physicians, as different specialties may be associated with different risks of exposure to SARS-CoV-2.

## Supporting information

S1 Checklist STROBE statement—Checklist of items that should be included in reports of cross-sectional studies. (DOC)
